# Stat3 Inhibits PTPN13 Expression in Squamous Cell Lung Carcinoma through Recruitment of HDAC5

**DOI:** 10.1155/2013/468963

**Published:** 2013-09-26

**Authors:** Xiu-juan Han, Li Xue, Li Gong, Shao-jun Zhu, Li Yao, Shu-mei Wang, Miao Lan, Wei Zhang, Yan-hong Li

**Affiliations:** ^1^The Helmholtz Sino-German Research Laboratory for Cancer, Department of Pathology, Tangdu Hospital, The Fourth Military Medical University, Xi'an 710038, China; ^2^Department of Urology, The Second Affiliated Hospital, Xi'an Jiaotong University, Xi'an 710004, China; ^3^Department of Gynaecology and Obstetrics, Tangdu Hospital, The Fourth Military Medical University, Xi'an 710038, China

## Abstract

Proteins of the protein tyrosine phosphatase (PTP) family are known to be signaling molecules that regulate a variety of cellular processes including cell growth, differentiation, and apoptosis. PTPN13 (also known as FAP1, PTPL1, PTPLE, PTPBAS, and PTP1E), a putative tumor suppressor, is frequently inactivated in lung carcinoma through the loss of either mRNA or protein expression. However, the molecular mechanisms underlying its dysregulation have not been fully explored. Interleukin-6 (IL-6) mediated Stat3 activation is viewed as crucial for multiple tumor growth and progression. Here, we demonstrate that PTPN13 is a direct transcriptional target of Stat3 in the squamous cell lung carcinoma. Our data show that IL-6 administration or transfection of a constitutively activated Stat3 in HCC-1588 and SK-MES-1 cells inhibits PTPN13 mRNA transcription. Using luciferase reporter and ChIP assays, we show that Stat3 binds to the promoter region of PTPN13 and promotes its activity through recruiting HDAC5. Thus, our results suggest a previously unknown Stat3-PTPN13 molecular network controlling squamous cell lung carcinoma development.

## 1. Introduction

The nonreceptor protein tyrosine phosphatase, PTPN13 (also known as FAP1, PTPL1, PTPLE, PTPBAS, and PTP1E) has recently been considered as a putative tumor suppressor [1, 2]. For instance, PTPN13 gene mutations have been identified in colorectal, head and neck, and hepatocellular carcinoma [3–5]. Besides, reduced PTPN13 expression in breast cancer correlates with decreased survival in patients [6]. Moreover, decreased PTPN13 expression synergizes with an activated ErbB2 transmembrane mutation (mNeuNT), which further enhances tumor progression and invasion in vivo [7]. In lung carcinoma, PTPN13 gene is frequently inactivated through the loss of either mRNA and protein expression or somatic mutation [8]. Although substantial advances have been made in understanding the mechanisms that regulate its expression, the molecular mechanisms by which PTPN13 is down-regulated in lung carcinomas remain largely unexplored.

Recent evidence has demonstrated that aberrant Stat3 signaling by Interlukin-6 (IL-6) in cancer cells is a major mechanism for tumor initiation, development, progression, and metastasis [9–11]. Stat3 is a transcription factor that can promote oncogenesis, and it is commonly activated in various types of cancer [12, 13]. Therefore, we speculate whether or not Stat3 activation could regulate PTPN13 expression in squamous lung carcinoma. Here, we show that mRNA and protein levels of PTPN13 are markedly reduced in HCC-1588 and SK-MES-1 cells treated with IL-6. We also suggest that Stat3 activation down-regulates PTPN13 expression through recruitment of HDAC5. Our findings, thus, link Stat3 signaling directly with the PTPN13 pathway, which have profound biological and therapeutic implications for squamous lung carcinoma.

## 2. Material and Methods

### 2.1. Cell Culture and Reagents

HCC-1588 and SK-MES-1 cells were purchased from the American Type Culture Collection (ATCC, USA) and Cell Bank of Type Culture Collection of the Chinese Academy of Sciences (CAS, Shanghai, China), respectively. Cells were cultured in Dulbecco modified Eagle's medium supplemented with 10% fetal calf serum (Gibco, Shanghai, China), 100 IU/mL penicillin (Gibco) and 100 mg/mL streptomycin (Gibco). IL-6 (Merck, Beijing, China) was added at a concentration of 20 ng/mL into cells at 60–80% confluence. 

### 2.2. Transient Transfections and Luciferase Reporter Assays

Human PTPN13 promoter was cloned into PGL3-basic plasmid (Promega, Madison, Wisconsin, USA). All the transient transfections were performed by Lipofectamine 2000 (Invitrogen, Shanghai), according to the manufacturer's instructions. The rate of Lip2000, vector was 1 : 300 (*μ*L : ng). For luciferase reporter assays, SK-MES-1 cells were seeded in 24-well plates and cotransfected with 200 ng PTPN13 promoter plasmids and 400 ng Stat3 expression plasmids or empty vectors (EV). Cells were harvested 36 hr after transfection. Luciferase activity was measured using the Dual Luciferase Reporter Assay System (Promega, USA).

### 2.3. RNA Extraction and Real-Time PCR Analysis

Total RNAs were extracted from cells by TRIzol reagent, and reverse transcriptions were performed by Takara RNA PCR kit (Takara, China) following the manufacturer's instructions. In order to quantify the transcripts of the interest genes, real-time PCR was performed using a SYBR Green Premix Ex Taq (Takara, Japan) on ABI 7900 (ABI, USA). PCR conditions included an initial holding period at 95°C for 5 minutes, followed by a two-step PCR program consisting of 94°C for 5 seconds and 60°C for 30 seconds for 45 cycles. *β*-actin was used as a reference gene. The primer sequences were listed as follows: PTPN13 (Forward: 5′-TTGGAATGACACTGTATTGGGG-3′, Reverse: 5′-CCAAGCAGTATGCTGTTGAGAT-3′), HDAC5 (Forward: 5′-GGTGTGGTCTACGACACGTTC-3′, Reverse: 5′-GATCCGCTCGCACTTGCTAA-3′), *β*-actin (Forward: 5′-CATGTACGTTGCTATCCAGGC-3′, Reverse: 5-CTCCTTAATGTCACGCACGAT-3′).

### 2.4. Western Blot

Cells were lysed in radioimmunoprecipitation (RIPA) buffer containing 50 mM Tris-HCl, 150 mM NaCl, 5 mM Mgcl_2_, 2 mM EDTA, 1 mM NaF, 1% NP40, and 0.1% SDS. Protein extracts were equally loaded on to 10–12% SDS-PAGE electrophoresed and transferred to nitro cellulose (NC) membranes (Amersham Bioscience, Buckinghamshire, UK). After blocking with 5% nonfat milk in PBS, the membranes were probed with antibodies followed by horseradish peroxidase-conjugated secondary antibodies. The signals were detected by chemiluminescent substrate kit (Millipore Corporation, Billerica, MA). The following antibodies were used: anti-Stat3 (Cell Signaling, Danvers, Massachusetts, USA), anti-PTPN13 (Abcam, Cambridge, Massachusetts, USA), anti-HDAC5 and GAPDH (Santa Cruz, California, USA). The GAPDH protein content was employed as a loading control.

### 2.5. Small Interfering RNA

All small interfering RNAs (siRNAs) were chemically synthesized by GenePharma (Shanghai, China). The siRNA sequences were as following: Stat3 (5′-CUGAUCACCUUUGAGACCGAGG-3′), HDAC5 (5′-CAGCUCCUGUUCGCUGAGUUC-3′). As negative control, a nonspecific sequence was used: 5′-CGUACGCGGAAUACUUCGA-3′. siRNA transfections were conducted using Lipofectamine 2,000 (Invitrogen) according to the manufacturer's instructions. Briefly, cells were seeded into 6-well plates and transfected with 60 nM siRNA oligos with 4 *μ*L Lipofectamine 2000 at 60–80% confluence.

### 2.6. ChIP Assays

A chromatin immunoprecipitation (ChIP) assay kit was used (Upstate, USA). In brief, SK-MES-1 cells were fixed with formaldehyde. DNA was sheared to fragments at 200–1000 bp by several sonications. The chromatin were incubated and precipitated with antibodies against human Stat3 (Cellsignaling), HDAC1 (Abcam), HDAC2 (Abcam), HDAC3 (Santa Cruz), HDAC5 (Santa Cruz) or IgG (Santa Cruz). The primer for real-time PCR covered the potential Stat3 binding site. The sequences were listed as following: (Forward: 5′-CGACTTACGACGACATTAC-3′, Reverse: 5′-TGACTAGGCATGCAATCGA-3′). 

### 2.7. Statistical Analysis

Values were shown as mean ± SEM. Statistical differences were determined by a Student's *t* test. Statistical significance is displayed as *(*P* < 0.05), **(*P* < 0.01) or ***(*P* < 0.001). 

## 3. Results

### 3.1. Down-Regulation of PTPN13 by IL-6 Treatment or Stat3 Activation

**Figure 1:**
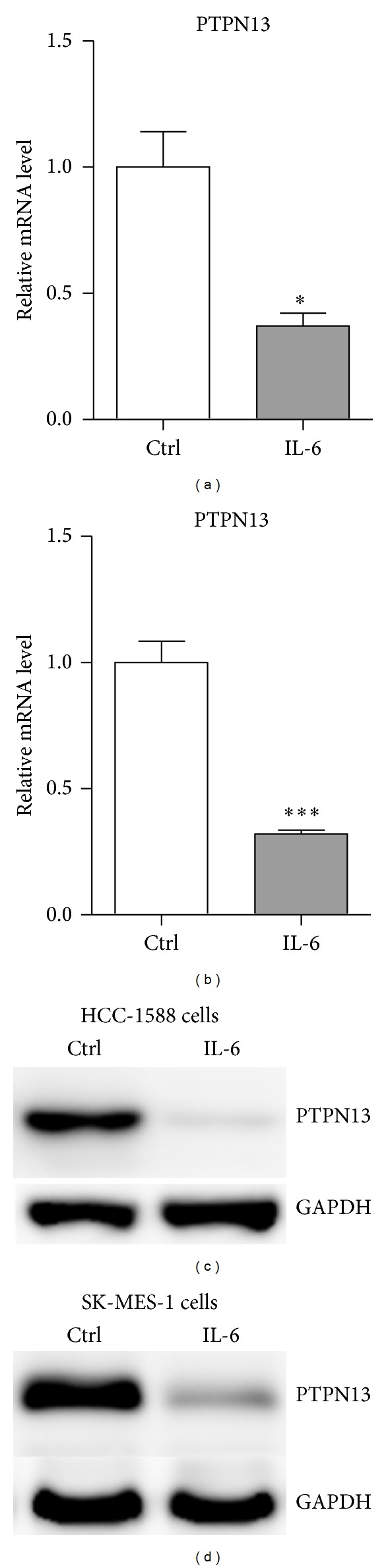
Down-regulation of PTPN13 by IL-6 treatment. ((a)-(b)) mRNA levels of PTPN13 were analyzed by real-time PCR in HCC-1588 (a) and SK-MES-1 (b) cells treated with vehicle control (Ctrl) or IL-6 (20 ng/mL). ((c)-(d)) Protein levels of PTPN13 were analyzed by western blot in HCC-1588 (c) and SK-MES-1 (d) cells treated with vehicle control (Ctrl) or IL-6 (20 ng/mL).

**Figure 2:**
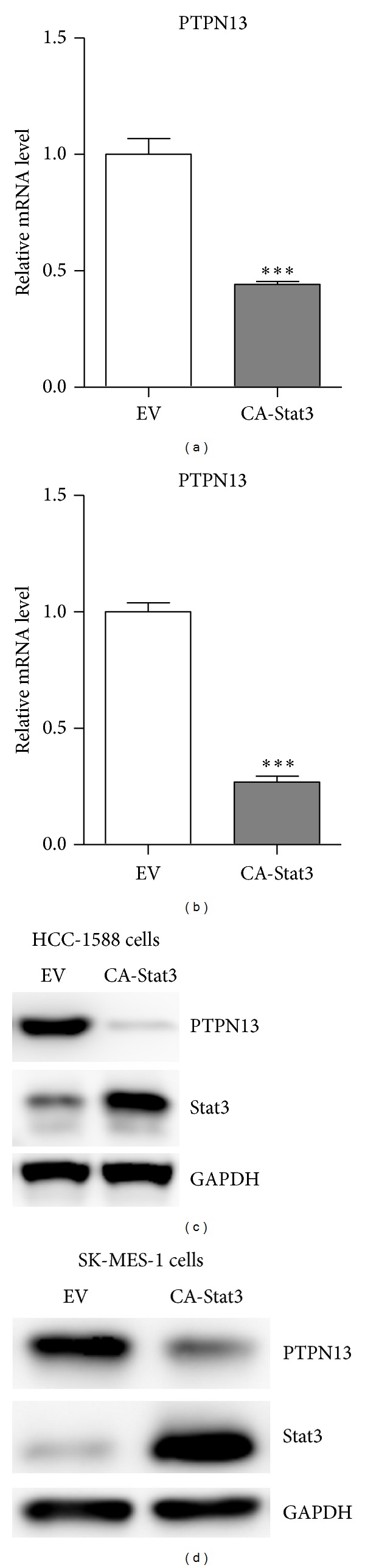
Down-regulation of PTPN13 by Stat3 overexpression. ((a)-(b)) mRNA levels of PTPN13 were analyzed by real-time PCR in HCC-1588 (a) and SK-MES-1 (b) cells transfected with empty vector (EV) or constitutive activated Stat3 (CA-Stat3). ((c)-(d)) Protein levels of PTPN13 were analyzed by western blot in HCC-1588 (c) and SK-MES-1 (d) cells transfected with empty vector (EV) or constitutive activated Stat3 (CA-Stat3).

Firstly, we used Real-time PCR and western blot to confirm the correlation between Stat3 activity and PTPN13 expression using two squamous lung carcinoma cells. As shown in Figures 1(a) and 1(b), IL-6 treatment significantly reduced PTPN13 mRNA levels in HCC-1588 and SK-MES-1 cells. Besides, its protein levels were also decreased in cells treated with IL-6 (Figures 1(c) and 1(d)). Moreover, overexpression of a constitutively activated Stat3 (Stat3C) [14], also reduced the expression of PTPN13 in both cells (Figures 2(a)–2(d)). 

### 3.2. Stat3 Inhibition by siRNA Oligos Increased PTPN13 Expression

**Figure 3:**
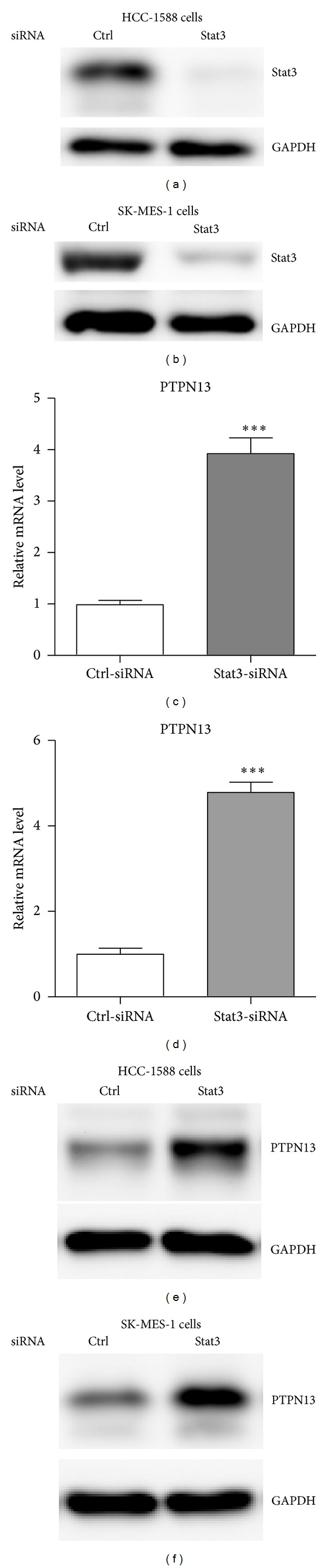
Stat3 knockdown up-regulates PTPN13 expression. ((a)-(b)) HCC-1588 and SK-MES-1 cells were transfected for nonspecific control (Ctrl) or Stat3 siRNA. Endogenous protein levels of Stat3 were determined. ((c)-(d)) mRNA levels of PTPN13 were analyzed by real-time PCR in HCC-1588 (c) and SK-MES-1 (d) cells transfected for nonspecific control (Ctrl) or Stat3 siRNA. ((e)-(f)) Protein levels of PTPN13 were analyzed by western blot in HCC-1588 (e) and SK-MES-1 (f) cells transfected for nonspecific control (Ctrl) or Stat3 siRNA.

Next, endogenous Stat3 expression was silenced by small interfering RNA (siRNA) oligos (Figures 3(a) and 3(b)). As a result, knockdown of Stat3 increased PTPN13 mRNA and protein levels in HCC-1588 and SK-MES-1 cells (Figures 3(c)–3(f)). Together, these data suggest that Stat3 could be an important upstream regulator in the control of PTPN13 expression in squamous lung carcinoma cells.

### 3.3. Stat3 Inhibits PTPN13 Expression through Being Bound to Its Promoter Region

**Figure 4:**
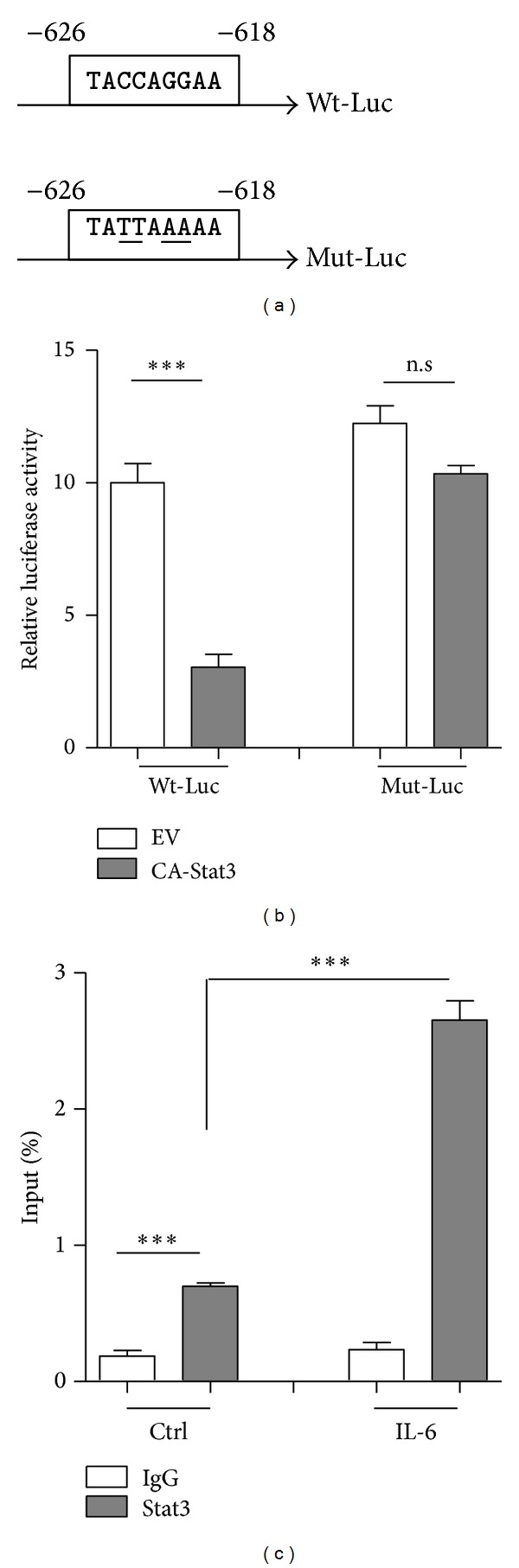
Stat3 inhibits PTPN13 expression through being bound to its promoter region. (a) The PTPN13 promoter constructs containing a potential Stat3 binding motif (−626 to −618) (Wild type: Wt-Luc). Point mutations underlined were induced in the Stat3 motif (Mutant: Mut-Luc). (b) SK-MES-1 cells were cotransfected with the indicated plasmids for 36 h, and the luciferase activity was measured. (c) Two antibodies (antiIgG and -Stat3) were used in the ChIP assays using SK-MES-1 cells. Cells were incubated with vehicle control (Ctrl) or IL-6 (20 ng/mL) for 2 hr. Cells were then subjected to ChIP analysis and quantified by real-time PCR.

We next determined whether Stat3 could be a transcriptional regulator of the PTPN13 gene. We analyzed the promoter of PTPN13 and identified a Stat3 motif located at −626 to −618 bp using an online promoter scanning system (http://www.cbil.upenn.edu/cgi-bin/tess/tess) (Figure 4(a)). SK-MES-1 cells were then transfected with a reporter vector encoding luciferase under the control of the PTPN13 promoter (WT-Luc). Concurrent expression of Stat3C with the PTPN13 reporter construct reduced PTPN13 promoter activity (Figure 4(b)), which was abrogated by the mutation of the Stat3 DNA-binding site in the PTPN13 promoter (Mut-Luc). We next carried out chromatin immunoprecipitation (ChIP) assays to assess whether Stat3 directly binds the PTPN13 promoter. Indeed, Stat3 protein could bind the PTPN13 promoter, which was significantly increased by IL-6 treatment (Figure 4(c)).

### 3.4. Stat3 Represses PTPN13 Expression through Recruitment of HDAC5

**Figure 5:**
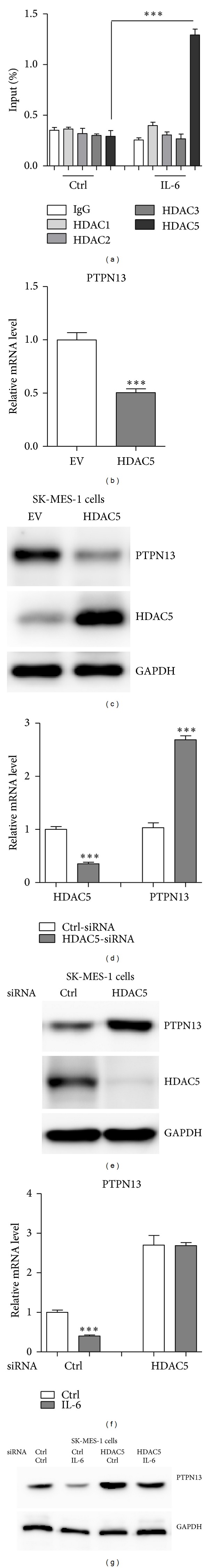
Stat3 represses PTPN13 expression through the recruitment of HDAC5. (a) Antibodies (antiIgG and -HDAC1, -HDAC2, -HDAC3, and -HDAC5) were used in the ChIP assays in SK-MES-1 cells. Cells were incubated with vehicle control (Ctrl) or IL-6 (20 ng/mL) for 2 hr. Cells were then subjected to ChIP analysis and quantified by real-time PCR. ((b)-(c)) mRNA and protein levels of PTPN13 were analyzed by Real-time PCR and western blot in SK-MES-1 cells transfected with empty vector (EV) or HDAC5. ((d)-(e)) SK-MES-1 cells were transfected for nonspecific control (Ctrl) or HDAC5 siRNA. Endogenous mRNA and protein levels of HDAC5 and PTPN13 were measured. ((f)-(g)) PTPN13 expression was analyzed by real-time PCR and western blot in SK-MES-1 cells treated with vehicle control (Ctrl) or IL-6 (20 ng/mL). Cells were pretransfected with nonspecific control (Ctrl) or HDAC5 siRNA for 24 hr.

Negative regulation of gene transcription by Stat3 has been partly attributed to the recruitment of corepressors such as the HDAC family [15]. Indeed, using chromatin immunoprecipitation assays, we observed that HDAC5, but not HDAC1, HDAC2, or HDAC3 was highly recruited to the promoter region of PTPN13 in SK-MES-1 cells treated with IL-6 (Figure 5(a)). Besides, overexpression of HDAC5 reduced endogenous PTPN13 mRNA and protein levels (Figures 5(b) and 5(c)). Consistently, knockdown of HDAC5 using small interfering RNA also increased PTPN13 mRNA and protein levels (Figures 5(d) and 5(e)), suggesting that Stat3 recruited nuclear HDAC5 to confer its transcriptional repression roles. In addition, HDAC5 deficiency attenuated the repressive roles of IL-6 treatment (Figures 5(f) and 5(g)), suggesting that HDAC5 is indispensable for the function of Stat3 to down-regulate PTPN13.

## 4. Discussion

In the present study, we show that Stat3 regulates PTPN13 expression in two human squamous lung carcinoma cells. Activation of Stat3 inhibits PTPN13 expression while Stat3 silencing up-regulates PTPN13 expression. At the molecular level, we identified a potential Stat3 binding site in the promoter region of PTPN13 gene. Therefore, for the first time, our results indicate that Stat3 could be a negative regulator of PTPN13 in squamous lung carcinoma. Given that Stat3 signaling is usually activated in cancers, our findings suggest a potential mechanism for the down-regulation of PTPN13 in lung carcinoma. Besides, it would be interesting to further investigate whether or not Stat3 could regulate PTPN13 in other cancer cells. In addition, we found that Stat3 recruited HDAC5 to repress PTPN13 promoter. Indeed, knockdown of HDAC5 abolished the inhibitory roles of Stat3, suggesting that HDAC5 is required for this regulatory pathway.

The epigenetic control of genes expression by the HDAC family has been demonstrated to play critical roles in cancer initiation, progression, and metastasis [16]. HDAC5, the protein encoded by this gene, belongs to the class II histone deacetylase family [17]. Previous reports have implicated it as an important epigenetic regulator contributing to development, cell differentiation, and apoptosis [18]. HDAC5 deficient mice have reduced cardiac polypyrimidine tract binding protein (PTB), protein abundance, and HDAC5 inhibition in myocytes which cause a reduction in endogenous expression of cellular FLICE-like inhibitory protein (cFLIP) and caspase-dependent cleavage of PTB, suggesting its critical role during cardiac muscle development [18]. Besides, HDAC5 is required for maintenance of pericentric heterochromatin, and it controls cell-cycle progression [19]. Specific ablation of HDAC5 by RNA interference led to profound changes in the heterochromatin structure and slowed down ongoing replication forks. Besides, HDAC5 depletion resulted in enhanced sensitivity of DNA to DNA-damaging agents, suggesting that heterochromatin decondensation induced by histone HDAC5 silencing may enhance the efficacy of cytotoxic agents that act by targeting DNA [19]. Moreover, recent studies demonstrated that HDAC5 was up-regulated in several types of cancers, such as acute lymphoblastic leukemia, medulloblastoma, and breast cancer [20–22]. Consistently, high HDAC5 expression was significantly associated with poor overall survival, implicating it as a novel marker for risk stratification and its role in tumor cell growth [20–22]. 

Notably, several HDACs inhibitors were shown to inhibit cancer cell proliferation and/or induce apoptosis in vitro and in vivo [23]. Besides, preclinical studies demonstrated the efficacy of HDAC inhibitors as anticancer agents, especially when HDAC inhibitors were used in combination with other therapies [24]. Therefore, understanding the roles and mechanisms of HDACs in tumorigenesis will provide the rationale for the development of specific HDACs inhibitors as efficient anticancer drugs.

In summary, our results provide new insight into how Stat3 activation could influence the PTPN13 expression. These findings may have implications for developing therapeutics for squamous cell lung carcinoma.

## References

[1] Wang Z, Shen D, Parsons DW (2004). Mutational analysis of the tyrosine phosphatome in colorectal cancers. *Science*.

[2] Freiss G, Chalbos D (2011). PTPN13/PTPl1: an important regulator of tumor aggressiveness. *Anti-Cancer Agents in Medicinal Chemistry*.

[3] Laczmanska I, Sasiadek MM (2011). Tyrosine phosphatases as a superfamily of tumor suppressors in colorectal cancer. *Acta Biochimica Polonica*.

[4] Niu J, Huang Y-J, Wang L-E, Sturgis EM, Wei Q (2009). Genetic polymorphisms in the PTPN13 gene and risk of squamous cell carcinoma of head and neck. *Carcinogenesis*.

[5] Frau M, Simile MM, Tomasi ML (2012). An expression signature of phenotypic resistance to hepatocellular carcinoma identified by cross-species gene expression analysis. *Cellular Oncology*.

[6] Glondu-Lassis M, Dromard M, Lacroix-Triki M (2010). PTPL1/PTPN13 regulates breast cancer cell aggressiveness through direct inactivation of Src kinase. *Cancer Research*.

[7] Vermeer PD, Bell M, Lee K (2012). ErbB2, EphrinB1, Src kinase and PTPN13 signaling complex regulates MAP kinase signaling in human cancers. *PLoS ONE*.

[8] Scrima M, De Marco C, De Vita F (2012). The nonreceptor-type tyrosine phosphatase PTPN13 is a tumor suppressor gene in nonsmall cell lung cancer. *American Journal of Pathology*.

[9] Yu H, Jove R (2004). The stats of cancer—new molecular targets come of age. *Nature Reviews Cancer*.

[10] Yu H, Pardoll D, Jove R (2009). STATs in cancer inflammation and immunity: a leading role for STAT3. *Nature Reviews Cancer*.

[11] Grivennikov S, Karin E, Terzic J (2009). IL-6 and Stat3 are required for survival of intestinal epithelial cells and development of colitis-associated cancer. *Cancer Cell*.

[12] Hedvat M, Huszar D, Herrmann A (2009). The JAK2 inhibitor AZD1480 potently blocks Stat3 signaling and oncogenesis in solid tumors. *Cancer Cell*.

[13] Lin D, Cui Z, Kong L, Cheng F, Xu J, Lan F (2013). p73 participates in WWOX-mediated apoptosis in leukemia cells. *International Journal of Molecular Medicine*.

[14] Lee H, Herrmann A, Deng J-H (2009). Persistently activated Stat3 maintains constitutive NF-*κ*B activity in tumors. *Cancer Cell*.

[15] Tang Y, Luo Y, Jiang Z (2012). Jak/Stat3 signaling promotes somatic cell reprogramming by epigenetic regulation. *Stem Cells*.

[16] Song S-H, Han S-W, Bang Y-J (2011). Epigenetic-based therapies in cancer: progress to date. *Drugs*.

[17] Parra M, Verdin E (2010). Regulatory signal transduction pathways for class IIa histone deacetylases. *Current Opinion in Pharmacology*.

[18] Ye J, Llorian M, Cardona M (2013). A pathway involving HDAC5, cFLIP and caspases regulates expression of the splicing regulator polypyrimidine tract binding protein in the heart. *Journal of Cell Science*.

[19] Peixoto P, Castronovo V, Matheus N (2012). HDAC5 is required for maintenance of pericentric heterochromatin, and controls cell-cycle progression and survival of human cancer cells. *Cell Death and Differentiation*.

[20] Moreno DA, Scrideli CA, Cortez MAA (2010). Differential expression of HDAC3, HDAC7 and HDAC9 is associated with prognosis and survival in childhood acute lymphoblastic leukaemia: research paper. *British Journal of Haematology*.

[21] Milde T, Oehme I, Korshunov A (2010). HDAC5 and HDAC9 in medulloblastoma: novel markers for risk stratification and role in tumor cell growth. *Clinical Cancer Research*.

[22] Patani N, Jiang WG, Newbold RF, Mokbel K (2011). Histone-modifier gene expression profiles are associated with pathological and clinical outcomes in human breast cancer. *Anticancer Research*.

[23] Shabason JE, Tofilon PJ, Camphausen K (2010). HDAC inhibitors in cancer care. *Oncology*.

[24] Khan O, La Thangue NB (2012). HDAC inhibitors in cancer biology: emerging mechanisms and clinical applications. *Immunology and Cell Biology*.

